# Successful Treatment of Lung Calciphylaxis With Sodium Thiosulfate in a Patient With Sickle Cell Disease: A Case Report

**DOI:** 10.1097/MD.0000000000002768

**Published:** 2016-02-12

**Authors:** Romain Arrestier, Caroline Dudreuilh, Philippe Remy, Ghada Boulahia, Bouteina Bentaarit, Claire Leibler, Amir Adedjouma, Tomek Kofman, Marie Matignon, Dil Sahali, Roger Dufresne, Jean-Francois Deux, Charlotte Colin, Philippe Grimbert, Philippe Lang, Pablo Bartolucci, Bernard Maitre, Jeanne Tran Van Nhieu, Vincent Audard

**Affiliations:** From the Service de Néphrologie et Transplantation, Hôpitaux Universitaires Henri Mondor, Centre de référence maladie rare Syndrome Néphrotique Idiopathique, Institut Francilien de recherche en Néphrologie et Transplantation (IFRNT), AP-HP (Assistance Publique–Hôpitaux de Paris), Créteil, France (RA, CD, PR, GB, BB, CL, AD, TK, MM, DS, PG, PL, VA); Equipe 21, INSERM Unité 955 (RA, CD, PR, GB, BB, CL, AD, TK, MM, DS, PG, PL, VA); Centre de Dialyse des Nouvelles Eaux Vives, 97100 Basse-Terre, Guadeloupe (RD); Service d’Imagerie Médicale, Hôpitaux Universitaires Henri Mondor, APHP, UPEC, Créteil, France (J-FD); Service de Pneumologie, Hôpitaux Universitaires Henri Mondor, APHP, UPEC, Créteil, France (CC, BM); Centre de Référence des Syndromes Drépanocytaires Majeurs, Hôpitaux Universitaires Henri Mondor, APHP, UPEC, Créteil, France (PB); and Département de Pathologie, Hôpitaux Universitaires Henri Mondor, APHP, UPEC, Créteil, France (J-TVN), Créteil, France.

## Abstract

Calciphylaxis is a small vessel vasculopathy, characterized by medial wall calcification that develops in a few patients with chronic renal failure. The prognosis of skin calciphylaxis has improved considerably since the introduction of sodium thiosulfate (STS), but it remains unclear whether this therapy is effective against organ lesions related to calciphylaxis. Pulmonary calciphylaxis is a usually fatal medical condition that may occur in association with skin involvement in patients with end-stage renal disease.

We report here the case of a 49-year-old woman homozygous sickle cell disease patient on chronic hemodialysis with biopsy-proven systemic calciphylaxis involving the lungs and skin. On admission, ulcerative skin lesions on the lower limbs and bilateral pulmonary infiltrates on chest computerized tomography scan were the main clinical and radiological findings. Skin and bronchial biopsies demonstrated calciphylaxis lesions. The intravenous administration of STS in association with cinacalcet for 8 consecutive months led to a clear improvement in skin lesions and thoracic lesions on chest computerized tomography scan.

This case suggests for the first time that organ lesions related to calciphylaxis, and particularly lung injury, are potentially reversible. This improvement probably resulted from the combination of 3 interventions (more frequent dialysis, cinacalcet, and STS), rather than the administration of STS alone.

## INTRODUCTION

Calciphylaxis, or calcific uremic arteriolopathy, is a rare, life-threatening medical condition related to a mineral and bone disorder that occurs mostly in patients with end-stage renal disease (ESRD) on dialysis.^1,2^ Its prevalence among hemodialysis patients has been reported to be up to 4% and its mortality rate is 40% to 80% after diagnosis.^3–5^ Painful ischemic purpura lesions and necrotic ulceration, generally affecting the lower limbs, are the hallmark cutaneous manifestations of calciphylaxis.^2^ The pathophysiological processes involved in the development of calciphylaxis remain uncertain but some risk factors have been identified, including Caucasian ethnicity, being female, impaired calcium and phosphorus metabolism, warfarin treatment, diabetes mellitus, obesity, hypoalbuminemia, and prolonged dialysis.^6^ The main histological features of the skin are medial calcification with local inflammation and intimal hypertrophy leading to vessel obstruction and cutaneous necrosis.^4,7^ Although skin lesions are the main clinical manifestations of calciphylaxis, other organs, including skeletal muscle, the lungs, brain, eyes, pancreas, and digestive tract, may also develop calciphylaxis lesions.^2^ Pulmonary calciphylaxis (PC) is rare and probably underdiagnosed because it can occur in the absence of clinical manifestations, but is usually fatal when associated with pulmonary clinical symptoms of the disease.^8^ Promising treatments that may stabilize the disease or improve its outcome, such as sodium thiosulfate (STS), have recently emerged as the gold standard therapy for calciphylaxis.^9^ Nevertheless, the beneficial effect of this therapeutic approach remains to be determined in patients with PC. We report here the case of a 49-year-old female sickle cell disease (SCD) patient on hemodialysis, who was successfully treated by intravenous STS in association with cinacalcet for systemic calciphylaxis with skin and lung lesions.

## CLINICAL REPORT

**FIGURE 1:**
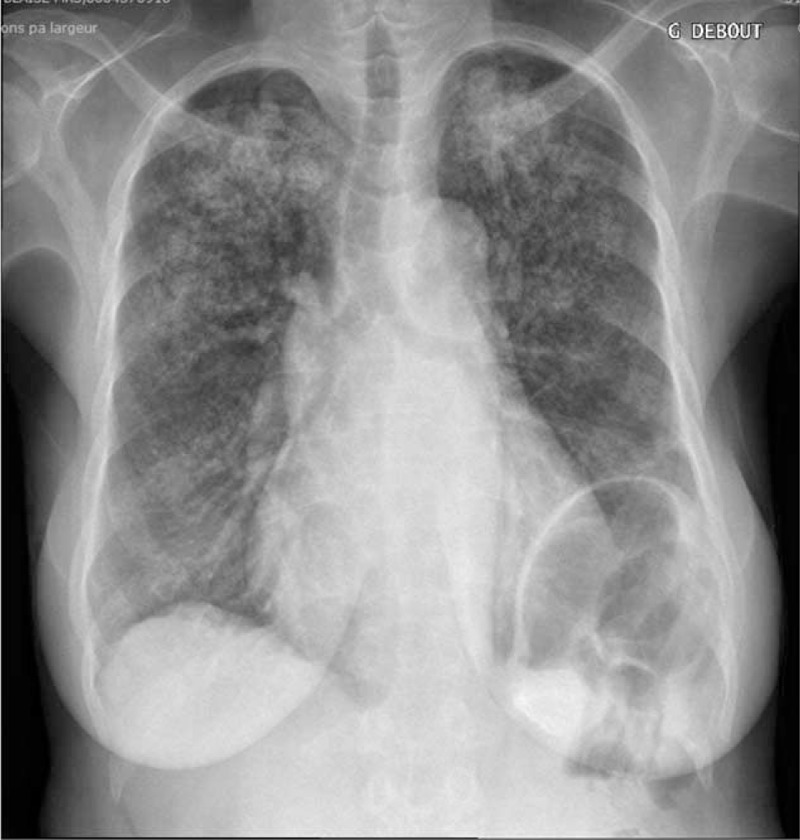
Chest x-ray at first clinical evaluation, initial chest x-ray showing diffuse calcifications, predominantly in the upper part of the lungs.

**FIGURE 2:**
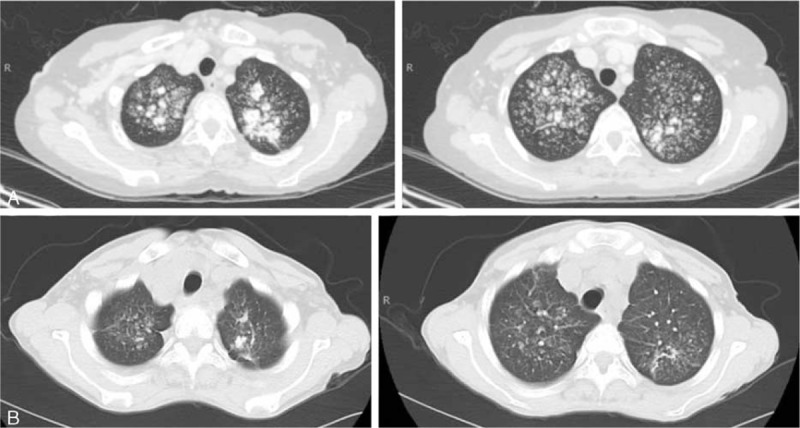
Chest CT scan at first clinical evaluation and 1 year later. A, Native CT examination at diagnosis, showing alveolar calcifications located predominantly in the upper lobes. B, One year later, after 8 consecutive months of sodium thiosulfate and cinacalcet therapy, CT examination revealed a regression of the lung calcifications. CT = computerized tomography

**FIGURE 3:**
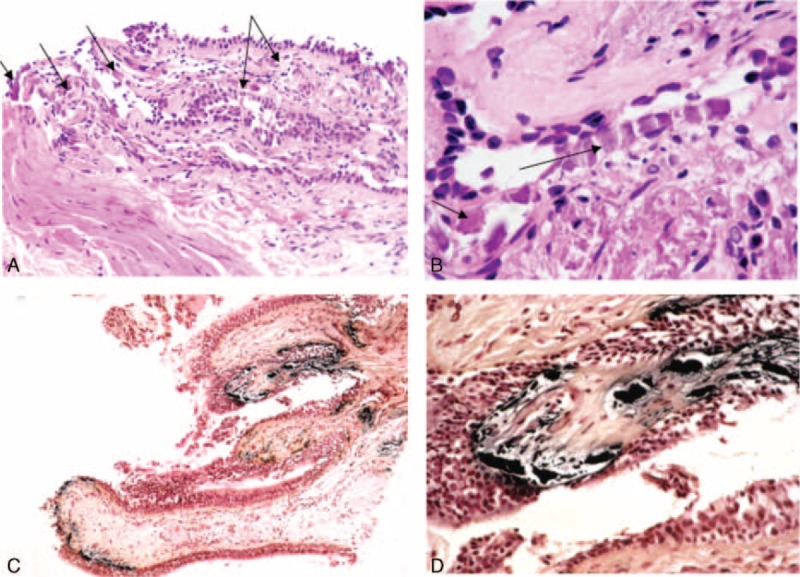
Bronchial biopsies providing evidence of calciphylaxis, bronchial biopsy specimens with abnormal subepithelial calcium deposits stained with hematoxylin and eosin (A and B, arrows, original magnification ×10 in both cases), and by the Von Kossa method (C and D, dark deposits, original magnification ×10 and ×40, respectively).

In July 2013, a 49-year-old woman from Caribbean origin with ESRD treated by chronic intermittent hemodialysis (initiated in 2008) was referred to our nephrology department for clinical evaluation before potential renal transplantation. Her relevant medical history included homozygous SCD with multiple vaso-occlusive crises and many episodes of acute chest syndrome. Chronic organ dysfunction related to SCD was observed, in the form of chronic kidney disease, post-transfusion iron overload and dilated cardiomyopathy with significant mitral insufficiency (stage 3). The patient had been on warfarin treatment since 2012 for paroxysmal atrial fibrillation. She was also on sevelamer, hydroxyurea, folic acid supplements, amiodarone, and ramipril, but was not taking calcium salt and vitamin D supplements. The patient received iron-chelating treatment (deferasirox) because of iron overload diagnosis. Physical examination on admission revealed multiple ulcerative skin lesions of 3 months’ duration on the lower limbs, but no definitive diagnosis was possible on the basis of the initial skin biopsy. Laboratory tests yielded the following results: serum calcium concentration = 2.54 mmol/L, serum phosphate concentration = 1.9 mmol/L, PTH concentration = 330 ng/L (normal range: 10–65 ng/L), albumin concentration = 33 g/L (normal range: 35–45 g/L) and CRP concentration = 27 mg/L. Hemoglobin level was 7.5 g/dL, and the patient had a white blood cell count of 4.2 × 10^9^/L (with a differential count of 2.6 × 10^9^ neutrophils/L and 1.4 × 10^9^ lymphocytes/L) and 291 × 10^9^ platelets/L. On admission, she had 4.2% HbF, 68.4% HbS, and 16.7% HbA1. She underwent transfusion with red blood cells every 6 weeks, approximately, to keep hemoglobin levels stable. The patient tested negative for antineutrophil cytoplasmic, antinuclear, and anti-DNA antibodies. Serum complement levels were within the normal range. Bone x-ray showed diffuse small artery and cutaneous calcifications without osteolytic lesions on the lower legs. A definitive diagnosis of calciphylaxis was made on the basis of a second skin biopsy showing subcutaneous small to medium-sized blood vessel calcifications associated with microthrombosis and intimal fibroplasia of panicular arterioles. Chest x-ray showed bilateral pulmonary infiltrates consisting of micronodules, predominantly at the top of the lungs (Figure 1). A thoracic CT scan revealed tree-bud opacities of the upper lobes associated with confluent alveolar opacities without intrathoracic adenopathy (Figure 2A). An accessory salivary gland biopsy provided no evidence of granuloma but was classified as Chisholm grade 1 (on a scale of 0–4). There were no clinical symptoms suggestive of lung involvement, but the results of the thoracic CT scan led us to perform bronchoalveolar lavage, which revealed a hypercellular fluid (with >9 × 10^5^ cells/mL) with a normal differential count (97% macrophages). Direct examination and cultures identified no viral, bacterial or parasitic pathogens. Bronchial biopsies showed a modified epithelium with the calcification of elastic fibers and calcium deposits in alveolar septal capillary walls, with moderate inflammatory infiltration, consistent with the diagnosis of PC (Figure 3). Pulmonary function tests showed that total lung capacity had decreased to 70% whereas pulmonary resistance had increased to 195%, suggesting restrictive pulmonary disease.

Systemic calciphylaxis was first treated by replacing warfarin with aspirin, increasing the frequency of dialysis (to 5 sessions per week) with a low calcium dialysate and providing nutritional support. Intravenous STS therapy was then initiated at a dose of 10 g 5 times per week for 4 months, progressively increasing to 12.5 g 5 times per week for an additional 4 months. In parallel, cinacalcet therapy (initial dose 60 mg/day) was initiated to limit hyperparathyroidism. Skin lesions did not require surgical wound debridement. Serum phosphate and calcium concentrations during treatment are summarized in Table 1. Six months after treatment initiation, the patient returned to the Caribbean. One year later, the patient was admitted to our department for clinical evaluation. A thoracic CT scan (Figure 2B) showed a substantial improvement in pulmonary lesions concomitant with a significant regression of skin lesions. This improvement in clinical and radiological calciphylaxis lesions was not associated with an improvement in pulmonary function test. Unfortunately, the patient died 3 months later, during hemodialysis, from a cardiac arrest of unknown cause.

**TABLE 1:**
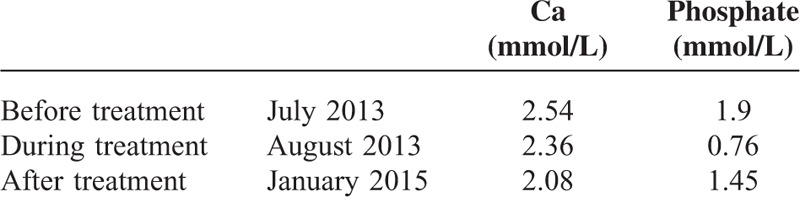
Serum Phosphate and Calcium Concentrations During Treatment

The authors did not obtain written consent of the patient because the patient died before we started to work on her case. The lung biopsy tissue collection was approved by the CNIL (Commission nationale d’informatique et des libertés).

## DISCUSSION

Skin manifestations are the predominant clinical lesions observed in patients with calciphylaxis.^4^ Our patient displayed cutaneous features of calciphylaxis, which were assessed by skin histology. Pulmonary calcifications on CT scan associated with calcium deposits in bronchial mucosa were highly suggestive of specific pulmonary involvement. The main calciphylaxis triggers identified in this case were warfarin and dialysis over a period of several years. In accordance with the advice of the cardiologist, we therefore decided to replace warfarin with aspirin, to limit the risk of further damage on warfarin treatment. Another factor potentially promoting calciphylaxis in this case was SCD, which is a well-known cause of endothelial dysfunction and leukocyte and platelet activation leading to chronic inflammation and hypercoagulation.^10^ Acute chest syndrome is a major cause of lung ischemia reperfusion injury and may also promote PC. Nevertheless, the few studies describing SCD patients with ESRD treated by dialysis did not focus on this specific complication.^11,12^ In our patient, the severity of calciphylaxis, including PC associated with skin lesions, led us to propose a multi-interventional curative approach involving the administration of both cinacalcet and STS. Cinacalcet has already been proposed as a promising treatment for calciphylaxisis.^2^ Further prospective controlled randomized studies are required to demonstrate a clear curative effect of STS, but this treatment has recently emerged as the cornerstone of calciphylaxis treatment.^2,9,13^ The optimal duration, mean dose and frequency of administration of STS were recently described by Nigwekar et al^14^ in a study of 172 patients. The mechanisms of action of STS remain elusive, although this drug is thought to increase the solubility of calcium phosphate through the formation of highly soluble calcium thiosulfate complexes and to have antioxidant and vasodilating properties.^2^ Pulmonary calciphylaxis is a rare complication in patients on dialysis and it is unclear whether STS is an effective treatment for this potentially fatal complication. Previous case reports of PC in patients on dialysis not receiving STS highlighted the potentially life-threatening nature of this medical condition, which frequently causes chronic respiratory failure or death.^15–18^ Cadavid et al^19^ described 1 patient with metastatic calfication and skin lesions suggestive of calciphylaxis in whom parenchymal infiltration was not significantly improved by STS and cinacalcet treatment. Nevertheless, the diagnosis of calciphylaxis in this patient was suspected on clinical grounds but not confirmed by biopsy, and no information was provided about the follow-up period. In our patient, after 1 year of follow-up, treatment with STS (administered for 8 months) in association with cinacalcet and an increase in dialysis frequency led to a significant improvement of parenchymal lung lesions, suggesting that chest injury is reversible with this therapeutic approach. This improvement probably resulted from the combination of 3 interventions (more frequent dialysis, cinacalcet, and STS), rather than the administration of STS alone. Nevertheless, our patient died for unknown reasons, possibly related to a thrombotic event due to warfarin withdrawal.

In conclusion, the findings for this case suggest that systemic organ damage, affecting the lungs in particular, should be systematically sought in patients with isolated apparent skin calciphylaxis. Sickle cell disease may be an additional risk factor for calciphylaxis, but this remains to be definitively demonstrated. Further studies are required to confirm the value of STS therapy for patients with PC, but this case strongly suggests that lung injury can be reversed by STS.
